# Schistosomiasis vaccine development: update on human clinical trials

**DOI:** 10.1186/s12929-020-0621-y

**Published:** 2020-01-22

**Authors:** Adebayo J. Molehin

**Affiliations:** 1 Center for Tropical Medicine and Infectious Diseases, Texas Tech University Health Sciences Center, 3601 4th Street, Lubbock, TX 79430 USA; 2 Department of Internal Medicine, School of Medicine, Texas Tech University Health Sciences Center, 3601 4th Street, Lubbock, TX 79430 USA

**Keywords:** Schistosomiasis, Neglected tropical disease, Control strategies, Mass drug administration (MDA), Schistosomiasis vaccine development, Clinical trials

## Abstract

Schistosomiasis causes significant levels of morbidity and mortality in many geographical regions of the world. The disease is caused by infections with parasitic blood flukes known as schistosomes. The control of schistosomiasis over the last several decades has been centered on the mass drug administration (MDA) of praziquantel (PZQ), which is the only drug currently available for treatment. Despite the concerted efforts of MDA programs, the prevalence and transmission of schistosomiasis has remained largely unchecked due to the fact that PZQ is ineffective against juvenile schistosomes, does not prevent re-infection and the emergence of PZQ-resistant parasites. In addition, other measures such as the water, sanitation and hygiene programs and snail intermediate hosts control have had little to no impact. These drawbacks indicate that the current control strategies are severely inadequate at interrupting transmission and therefore, implementation of other control strategies are required. Ideally, an efficient vaccine is what is needed for long term protection thereby eliminating the current efforts of repeated mass drug administration. However, the general consensus in the field is that the integration of a viable vaccine with MDA and other control measures offer the best chance of achieving the goal of schistosomiasis elimination. This review focuses on the present status of schistosomiasis vaccine candidates in different phases of human clinical trials and provide some insight into future vaccine discovery and design.

## Introduction

Although humans have been plagued with schistosomiasis since at least 1500 BC [1], it was not until the mid-1800s that the disease was first described by a German physician, Theodor Bilharz, during an autopsy [2]. Schistosomiasis is a neglected tropical disease caused by infections with parasitic trematode belonging to the genus *Schistosoma*. The infection is widespread in the tropics and subtropics and the most clinically-relevant species in terms of prevalence and disease caused are *Schistosoma mansoni*, *Schistosoma haematobium* and *Schistosoma japonicum* while *Schistosoma intercalatum, Schistosoma mekongi* and *Schistosoma guineensis* have lower global prevalence. In regions of endemicity, schistosomiasis exacts significant levels of human morbidity and mortality with an estimated 258 million people currently infected worldwide and an additional 779 million people at risk of infection. As standard methods for diagnosing schistosomiasis are quite insensitive, experts believe that actual estimates of the number of people currently infected with schistosomiasis ranges between 400 and 600 million based on the hypothesis that there is a one to one (1:1) ratio between egg-positive infected individual and an egg-negative infected individual [3]. Schistosomiasis is estimated to cause 280,000 deaths annually in 78 countries and about 3.8 million disability adjusted life years credited to the disease [4, 5].

**Fig. 1:**
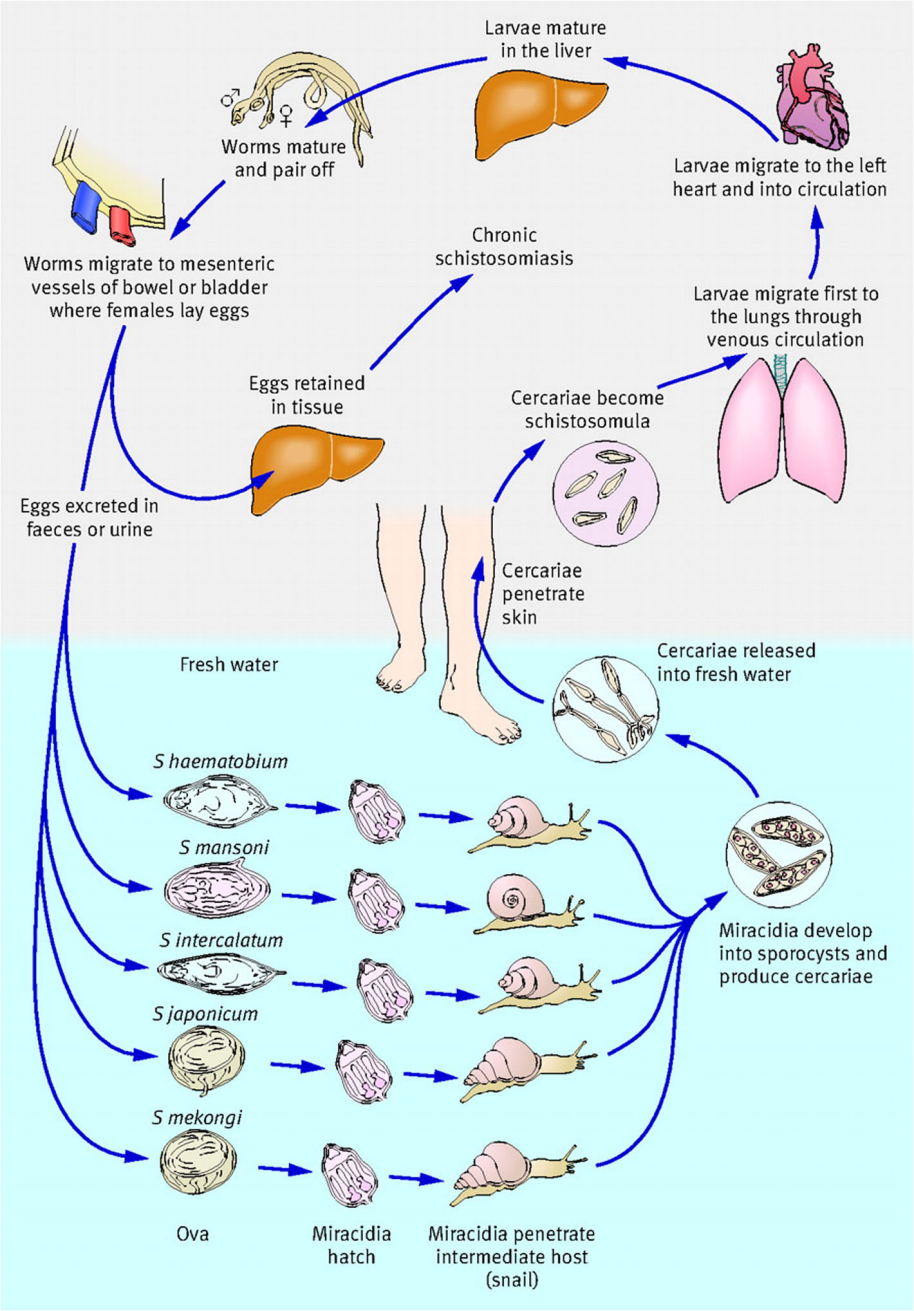
Life cycle of schistosomes. Five of the species of schistosome that infect humans are depicted. Infection occurs upon contact with fresh water contaminated with the free-swimming larvae known as cercariae. Cercariae penetrates the skin of humans and/or other mammalian hosts, shed their tails and transform into migrating larvae which enter into circulation traversing various host organs en route to the lungs. After many days, the worms exit the lungs and migrate to the veins of the portal system where they mature into adult male and female worms and form pairs. Worm pairs then migrate to the either the superior mesenteric veins (*Schistosoma mansoni*) inferior mesenteric and superior hemorrhoidal veins (*S. japonicum*), or the vesical plexus and veins draining the ureters (*S. haematobium*). Oviposition begins approximately 4–6 weeks post infection and continues throughout the lifespan of the worm. Some of the eggs laid pass from the lumen of blood vessels into various host tissues in close proximity while the remaining eggs pass through the intestinal wall or bladder and are released into the environment in feces (*S. mansoni* and *S. japonicum*) or urine (*S. haematobium*). Upon contact with water, the released eggs hatch into motile miracidia, which in turn, infect specific fresh water intermediate snail host depending on the species. *S. mansoni* infects *Biomphalaria* species while *S. haematobium* infects *Bulinus* species and *S. japonicum* infects *Oncomelania* species. Within the snail host, the larvae undergo a series of asexual reproduction and develop into sporocysts. Upon exposure to sunlight, ceracariae are released into fresh water to infect suitable mammalian hosts. Figure obtained from Gray DJ et al. [8] and used with permission

Infection occurs when the skin of a human host is penetrated by the free-swimming larvae, cercariae, released by various snail intermediate hosts upon contact with contaminated fresh water [6, 7]. The life cycle of schistosomes is illustrated in Fig. 1. Infections with *Schistosoma mansoni* cause hepatic/intestinal schistosomiasis in Brazil, sub-Saharan African, Puerto Rico, Venezuela, Republic of Suriname and the Caribbean islands while *S. haematobium* causes urogenital schistosomiasis in sub-Saharan African and the Middle East, namely Egypt, Sudan and Yemen [9]. *S. japonicum*, a zoonotic trematode, causing hepatic/intestinal disease (Asiatic or Oriental schistosomiasis) in the People’s Republic of China, the Philippines and Indonesia [10].

Since its development by Bayer in the 1970s, mass drug administration (MDA) of praziquantel (PZQ) [11] has been the main strategy for the control of schistosomiasis [12]. Despite the large-scale efforts at controlling schistosomiasis through the various MDA programs, the prevalence and intensity of schistosomiasis have remained unabated. Although PZQ is highly effective against adult schistosome parasites, its exclusive use as a monotherapy raises the concern of drug failure due to the possible emergence of drug-resistant parasites. Other drawbacks with PZQ includes its ineffectiveness against juvenile worms resulting in less desirable outcomes during MDA campaigns. In addition, PZQ does not prevent re-infection and substantial efforts and infrastructure are also required to achieve the coverage needed for effective schistosomiasis control [13]. Reports by the World Health Organization indicated 20.7% global coverage of mass PZQ administration in 2014 [14]. Other control measures such as intermediate snail host control, water, sanitation and hygiene (WaSH) programs have also had very little impact [15]. Despite this massive and concerted efforts, active transmission of schistosomiasis are now being reported in geographical areas formerly known to be free of schistosomiasis [16]. Given the predicaments already discussed, it is now apparent that in order for meaningful progress to me made towards sustainable control of schistosomiasis, integrated control measures are required with an effective vaccine playing a key role. In *Science* “Unfilled vials” feature, schistosomiasis vaccine was ranked 7th among top 10 ‘shots’ that require urgent development in order of R&D priority based on feasibility and need [17].

## A case for schistosomiasis vaccine

Historically, the deployment and use of vaccines have ranked the most cost-effective way for preventing diseases caused by infectious pathogens [4, 18]. In fact, it is not overreaching to imply that vaccination has possibly made the most significant contribution to global health following the introduction of clean water and proper sanitation. Although, there is currently no vaccine available for human use against schistosomiasis, strong evidence from human field studies and experimental animal models of schistosomiasis support the feasibility of developing of an effective vaccine for long term protection [19, 20]. In a nut shell, our current knowledge on the immunology of schistosomiasis can be divided into two main components: 1) immune responses to secreted egg antigens within the host tissues, particularly the liver and 2) age-dependent acquired immunity in schistosome-endemic areas resulting from years of exposure to the parasite.

Evidence from human field studies have shown that people living in endemic areas can develop partial immunity to infection. In fact, a small cohort of people living in Brazil, referred to as putative resistant, are naturally resistant to schistosomiasis, showing no clinical signs of the disease despite years of exposure [21]. Data from experimental animal models of schistosomiasis also provide a strong case for schistosomiasis vaccine development. Studies utilizing non-permissive hosts such as rats and rhesus macaques, have shown that resistance to schistosome infections and subsequent worm elimination is entirely host immune dependent [22, 23]. Furthermore, several vaccine efficacy studies in rodents and nonhuman primates using either radiation-attenuated (RA) cercariae or recombinant antigens as vaccines have shown promising results (reviewed in ref. (4, 13)). Although the complexity of schistosome life cycle poses a unique challenge at identifying good vaccine targets, on the other hand, it presents an opportunity to identify targets unique to particular life cycle stages. Perhaps, of utmost importance is the fact that schistosomes do not multiply within their definitive hosts (e.g. humans) thereby making even a vaccine-mediated partial worm burden reduction a huge benefit in terms of schistosomiasis control [24]. Field trials using veterinary-based vaccines against other parasitic helminth infections caused by *Taenia solium* [25] and *Echinococcus granulosus* [26] showed significant efficacy suggesting there is a reason for optimism with respect to developing an effective schistosomiasis vaccine. However, considerable research and development efforts are still needed if this is to become a reality sooner rather than later.

## Schistosomiasis vaccine development

Over the past few decades many working groups of experts have made recommendations concerning the type of schistosomiasis vaccine candidates that should be considered for development and what characteristics constitute an effective vaccine. The overall consensus from these working groups, developed as Preferred Product Characteristics (PPC) for a schistosomiasis vaccine, is that an effective prophylactic vaccine should reduce adult worm burden by 75% in immunized individuals and that the reduction in egg excretion rates by infected individual should be close to 75% as well. Other pertinent information on the schistosomiasis vaccine PPC have already been extensively reviewed elsewhere [4, 14].

Within the last few decades, more than one hundred candidate antigens have been identified and tested against one or more of the three main species of schistosomes infecting humans in various animal models of infection and disease. Although many of these antigens offered some protection, and are considered promising, only a handful candidates have made it to human clinical trials to date. One of the main reasons attributed for not having more antigens in clinical phase of development is the fact that most of the antigens identified were only evaluated in murine models with well documented intrinsic flaws [27]. For instance, over 68% of penetrating *S. mansoni* cercariae fail to mature into adult worms in naïve mice [27] thereby supporting the argument that the choice of appropriate animal models is critical to the development of schistosomiasis vaccine. It has been argued that in order to have the highest possible clinical efficacy, current or newly identified promising vaccine candidates should be tested in nonhuman primates [14]. In fact, experts are now strongly advocating that results of pre-clinical vaccine trials in baboons should be a prerequisite before any extensive scale-up to human trials is ever considered [27]. However, in addition to the huge costs of conducting research with these primates, justifying the use of these animals from ethics standpoint is also very challenging particularly, in schistosomiasis vaccine efficacy studies in which the animals have to be euthanized in order to comprehensively assess vaccine effectiveness. Nonhuman primates (e.g. baboons) are natural hosts of schistosomes with cercarial infections yielding close to 90% maturation into adult worms. The clinical manifestations in these animals are reflective of what happens in humans. Other benefits of the nonhuman primate model include similarity to human in terms of physiology and immune response [27]. Another key consideration in schistosomiasis vaccine development is the potential risk of candidate antigens inducing undesirable atopic IgE responses in immunized individuals as it was reported in the field trial of a hookworm vaccine, *Na*-ASP-2 [28].

## Schistosomiasis vaccines in human clinical trials

Of all the many candidate antigens that have been characterized and tested over the past 2–3 decades against one or more of the three main schistosome species, only three are currently in various phases of human clinical trials. These recombinant antigens include *Schistosoma haematobium* 28-kD glutathione S-transferase (rSh28GST) [29, 30], *Schistosoma mansoni* 14-kDa fatty acid-binding protein (Sm14) [31] and *Schistosoma mansoni* tetraspanin, a 9-kDa surface antigen (Sm-TSP-2) [32]. The fourth antigen which was only very recently approved for human clinical trial is the large subunit of the *Schistosoma mansoni* calpain (Sm-p80) [33]. Table 1 summarizes candidate vaccine formulations in different phases of clinical development.

**Table 1:** Schistosomiasis candidate vaccines in human clinical trials

Candidate Vaccine	Species targeted	Clinical Phase	Efficacy in humans and/or animal models	Sponsor
Recombinant Sh28GST/Alhydrogel® (Bilharvax)	*Schistosoma haematobium*	Phases 1, 2 & 3 completed	No protection in immunized humans. No effect on worm burden in immunized monkeys but 50% reduction in tissue egg load and up 77% reduction in excreted eggs	University Hospital, Lille & Institut National de la Santé Et de la Recherche Médicale
Recombinant Sm14/GLA-SE	*Schistosoma mansoni*	Phases 1 & 2a completed. Phase 2b initiated	67 and 93% worm reduction in immunized mice and rabbits respectively.	Oswald Cruz Foundation
Recombinant Sm-TSP-2/ Alhydrogel®	*Schistosoma mansoni*	Phase 1a completed. Phase 1b initiated	Immunized mice had 57 and 64% reduction in worm and liver egg burden respectively	Baylor College of Medicine
Recombinant Sm-p80/GLA-SE	*Schistosoma mansoni*	Phase 1 initiated	93% reduction in adult female worms in immunized baboons. 90% reduction in tissue egg load and 81% reduction in egg hatching rate	Texas Tech University Health Sciences Center

### *Schistosoma haematobium* 28-kD glutathione S-transferase

The 28-kDa glutathione S-transferases (28GST) are enzymes involved in parasite metabolic processes and host immune modulation. In schistosomes, 28GST have been shown to abrogate the movement of epidermal Langerhans cells to the draining lymph nodes [34]. In schistosomes, 28GSTs are also known to functionally bind testosterone [35] and are key enzymes involved in parasite detoxification pathways [36]. Since its development and characterization [30], *Schistosoma haematobium* 28-kD glutathione S-transferase (rSh28GST) has been extensively tested as potential schistosomiasis vaccine in various animal models. In a nonhuman primate efficacy study, rSh28GST showed no vaccine effect on worm burden but had a profound effect on tissue egg load and fecal egg excretion [29]. It is important to note that this study was conducted using patas monkeys (*Erythrocebus patas*) which appeared not be a good host for *Schistosoma haematobium* with adult worm recovery rate of less than 4% from the control group [29]. A phase 1 human clinical trial showed that rSh28GST adsorbed to Alhydrogel induced a Th2-dependent immune response in immunized healthy subjects and was well tolerated [37]. The same authors also reported that the co-administration of rSh28GST and PZQ was also well tolerated in healthy volunteers during a Phase 2 clinical trial [38]. However, in a recent phase 3 trial design to investigate the safety, immunogenicity and efficacy of rSh28GST in infected children, no sufficient efficacy was observed. Some of the reasons suggested by the authors for lack of efficacy were possible counter effects of repeated PZQ administration and vaccine regimen geared towards blocking IgG4 production rather than induction of protective IgG3 antibodies [38].

### *Schistosoma mansoni* 14-kDa fatty acid-binding protein

Schistosomes utilize fatty acid-binding proteins (FABP) for the uptake, transport and compartmentalization of host-derived sterols and fatty acids since they are completely deficient in the oxygen-dependent pathways needed for the de novo synthesis of these essential organic molecules [39]. Since its identification in *Schistosoma mansoni* (Sm14), FABPs have now been characterized in several other helminths. Due to the critical function play by these proteins, Sm14 was regarded as a potential schistosomiasis vaccine candidate [39]. Efficacy testing using recombinant Sm14 (rSm14) without any adjuvant showed a remarkable 67% protection reduction in adult worm burden in outbred Swiss mice and up to 93% protection in New Zealand white rabbits following *S. mansoni* cercarial challenge [40]. Sm14 was also shown to confer cross-species protection against *Fasciola hepatica* infections in mice [40] and sheep [41]. A phase 1 clinical trial in healthy volunteers from non schistosome endemic area using Sm14 formulated in glucopyranosyl lipid A adjuvant in an oil-in-water emulsion (GLA-SE) (Sm14/GLA-SE) was successfully conducted. Results showed that Sm14/GLA-SE was well tolerated in the human subjects with few mild adverse events. Immunization with Sm14/GLA-SE elicited robust Sm14-specific humoral immune responses with no production of deleterious IgE antibodies [42]. The clinical phase 2a trial designed to assess the safety and immunogenicity of Sm14/GLA-SE in health male adults living in highly endemic area was concluded in 2017 (https://clinicaltrials.gov/ct2/show/NCT03041766). However, the results from the study are yet to be published. Further immunogenicity and safety studies (Sm14 Phase 2b-Sn) in infected school-aged children have been approved and should be commencing soon (https://clinicaltrials.gov/ct2/show/NCT03799510).

### *Schistosoma mansoni* tetraspanin

Tetraspanins (TSP) are a family of proteins that are abundantly expressed on the schistosome surface membrane [43, 44], and therefore are exposed to the host immune system. The two main tetraspanins in *S. mansoni* are TSP-1 and TSP-2 termed Sm-TSP-1 and Sm-TSP-2 respectively [45]. Structurally, tetraspanins are made up of four transmembrane domains linked by two extracellular loops - a short and a longer loop [45]. Efficacy testings have focused on using the extracellular loop of the tetraspanin because it is the portion that is readily accessible to the host immune system. Due to the fact that only TSP-2 (and not TSP-1) was strongly recognized by protective antibodies (IgG1 and IgG3) from naturally immune individuals (putative resistant) and not by antibodies from chronically infected or naïve individuals, efficacy testings leading to clinical trials have therefore focused on the TSP-2 antigen [45]. Mouse studies have shown that rSm-TSP-2 offered significant protection against *S. mansoni* challenge infections as demonstrated by 57 and 64% reduction in worm and liver egg burdens respectively [45]. Although the phase 1a clinical trial of Sm-TSP-2/Alhydrogel® was initiated in 2014 and possibly completed, the results from the study are yet to be published. However a phase 1b dose-escalation study to evaluate the safety, reactogenicity and immunogenicity of Sm-TSP-2/Alhydrogel® with or without AP 10–701 in health exposed adults has been recently initiated with the expected completion date in June 2023 (https://clinicaltrials.gov/ct2/show/NCT03910972).

### *Schistosoma mansoni* calpain

Calpain is a calcium activated neutral protease abundantly expressed on the tegument of adult schistosomes as well as in the various life cycle stages of the parasite [46]. Sm-p80 is the large subunit of *S. mansoni* calpain and it plays a major role in the tegument biogenesis and/or turnover, a mechanism employed by blood-dwelling helminths to evade and/or modulate their host immune responses [33]. Due to the critical role of Sm-p80 in the survival of schistosomes within their hostile host environment, it was developed as a potential schistosomiasis vaccine candidate. Several efficacy studies have been conducted over the last two decades using Sm-p80-based vaccines. Data from several murine and nonhuman primate studies showed prophylactic, therapeutic, anti-pathology and transmission-blocking efficacies as demonstrated by significant reduction in adult worms, killing of established worms [47], reduction in tissue egg load and fecal egg excretion [14]. Recently, in a double-blind preclinical trial, baboons immunized with Sm-p80/GLA-SE had a 93.45% reduction in adult female worms with a consequent 89.95% reduction in tissue egg load [48]. The authors also reported a significant reduction in hatching rates of eggs excreted in feces [48]. In another recent study designed to mimic field scenario of schistosomiasis vaccine deployment, it was shown that Sm-p80-based vaccination following PZQ treatment of chronically-infected baboons caused a significant reduction in tissue egg retention and hatching rates when subsequently challenged with *S. mansoni* cercariae [49]. Notably, infected human populations tested to date have not been shown to express Sm-p80-specific IgE antibodies [50, 51] thereby lessening the potential risk of severe allergic reaction. Sm-p80/GLA-SE vaccine (SchistoShield®) has now been approved for Phase 1 clinical trials scheduled to begin in early-mid 2020.

## Concluding remarks

Schistosomiasis remains a major global health concern in many parts of the world. With the inadequacies/limitations of the current control approaches centered on mass PZQ treatment, the development and deployment of an effective schistosomiasis vaccine as part of an integrated program is to be highly promoted. Our current understanding of the immunity against schistosomiasis has largely come from studies in mice, therefore caution should be exercised in extrapolating data obtained from murine models for human clinical trials. The growing concerns regarding the unsuitability of mouse models to assess the efficacy of candidate antigens further supports the argument that using more suitable animal models such as nonhuman primates for *S. mansoni* and *S. haematobium* and bovines for *S. japonicum* is clearly necessary. Since only a handful of candidates are in clinical trials, the discovery of more candidate antigens cannot be overemphasized. While the development of schistosomiasis vaccine has proven to be very challenging, there are still reasons to be optimistic. With the growing field of systems vaccinology, vaccinomics, immunomics and technologies such as RNA interference and CRISPR/Cas9, an effective vaccine will be a reality sooner rather than later.

## Data Availability

Not applicable.
